# Potentialities and Limits of Some Non-thermal Technologies to Improve Sustainability of Food Processing

**DOI:** 10.3389/fnut.2018.00130

**Published:** 2019-01-17

**Authors:** Laetitia Picart-Palmade, Charles Cunault, Dominique Chevalier-Lucia, Marie-Pierre Belleville, Sylvie Marchesseau

**Affiliations:** ^1^IATE, University of Montpellier, CIRAD, INRA, Montpellier SupAgro, Montpellier, France; ^2^IEM, University of Montpellier, CNRS, ENSCM, Montpellier, France

**Keywords:** non-thermal technologies, sustainability, food processing, high pressure, membrane processes

## Abstract

In the whole food production chain, from the farm to the fork, food manufacturing steps have a large environmental impact. Despite significant efforts made to optimize heat recovery or water consumption, conventional food processing remains poorly efficient in terms of energy requirements and waste management. Therefore, in the few last decades, much research has focused on the development of alternative non-thermal technologies. Some of them, such as membrane separation processes, hydrostatic or dynamic high pressure, dense phase or high-pressure carbon dioxide, and pulsed electric fields (PEFs) have been extensively studied for cold pasteurization, concentration, extraction, or food functionalization. However, it is still difficult to evaluate the actual advantages or limits of these innovative processing technologies to replace conventional processes. Thus, the overall aim of this paper is to present an overview of the most relevant studies dealing with the potentialities and limits of these non-thermal technologies to improve sustainability of food processing. After a brief presentation of the physical principles of these technologies, the paper illustrates how these technologies could play a decisive role for sustainable food preservation or valorization of raw materials and by-products.

## Introduction

As clearly expressed in the 2030 agenda for sustainable development of United Nations, the global food system is directly or indirectly linked to most of the sustainable development goals proposed to promote and plan sustainable development worldwide (1). The challenge in the coming decades will be to ensure the availability of sufficient safe, nutritious, tasty and convenient food to the rapidly expanding and more affluent population while achieving sustainability (2, 3). At the present time, the global food system, composed of many sequential steps from agricultural production to consumers, is characterized by different sustainable weaknesses which still remained evaluated from limited number of common indicators, even if some recent studies proposed now multi-indicator analysis (4). The environmental impact of agricultural production was particularly investigated, showing among other things, that it is responsible for ~1/4 of all greenhouse gas emissions from human activities (5). In addition, ~25% of water consumption worldwide was attributed to food processing, and it is also responsible for the highest contribution to the emissions of organic water pollutants (6, 7). Moreover, ~30–50% of produced foods become waste (8), fruits, vegetables, cereals, and cereal products contribute the most to the food loss and waste throughout the food supply chain (9). Aquatic, atmospheric and solid waste generation characterizes the impact of food processing on the environment, and the improvement initiatives are consequently shift toward three main axes: energy consumption, solid waste reduction or up-cycling, water consumption, and wastewater reduction. Obviously, other drivers influence the efforts engaged: environmental legislation has to be respected and complex consumer choice and preference has to be foreseen before implementing alternative products or services (10). Besides, the increase in energy prices typically leads food manufacturing companies to invest in a better energy management. With respect to the latter point, thermal processes (pasteurization, sterilization, evaporation, refrigeration, freezing, and drying) are characterized as the most energy-consuming technologies in the food industry. But the conventional thermal processes are directly in line with one of the priorities concerning food processing: food safety—which requires processing steps to decrease microbial loads and consequently enhance safety and shelf life.

The development of green technologies in the food manufacturing sector is particularly relevant with the objective to convert raw agro materials into food products with the desired quality and functional properties while increasing manufacturing efficiency. In addition, companies will have to remain competitive at a time when consumer and government demands for sustainable development are constantly increasing (11, 12). Specifically, non-thermal processes recognized as value-added technologies have gained importance the last few decades as sustainable alternatives to conventional food processing—through direct reduction of energy and water consumption during processing, but also by reducing energy impact during storage. The indirect effects of non-thermal processing are also expected as a contribution to solid waste reduction and valorization of biomass resources (11, 13). The indirect impact of non-thermal processing on food processing sustainability could be even larger than direct impacts, since food losses, suboptimal utilization of by-products/processing residues and unnecessary quality decay within the supply chain are major inefficiencies within the food manufacturing sector.

Several emerging high-potential technologies, including high pressure (high hydrostatic pressure (HHP) and dynamic high pressure), pulsed electric field (PEF), and carbon dioxide processing, as well as membrane processing (which is already well-established), are all discussed in this paper to illustrate the potential impact of non-thermal food processing technique on improving the sustainability of food processing operations. These technologies are based on physical or chemical constraints, and have the particularity to be efficient at mild temperatures compared to conventional food processing operations used in industry to stabilize food products or extract compounds of interest (14).

The purpose of this review is to provide an overview of the current status and trends dealing with the potentialities and limits of these selected non-thermal technologies. The review intends to present and compare these different technologies according to three applications closely linked to food processing sustainability: stabilization, extraction and water recovery, and food waste management.

## Overview Of Principles And Technologies

### High Hydrostatic Pressure

**Figure 1:**
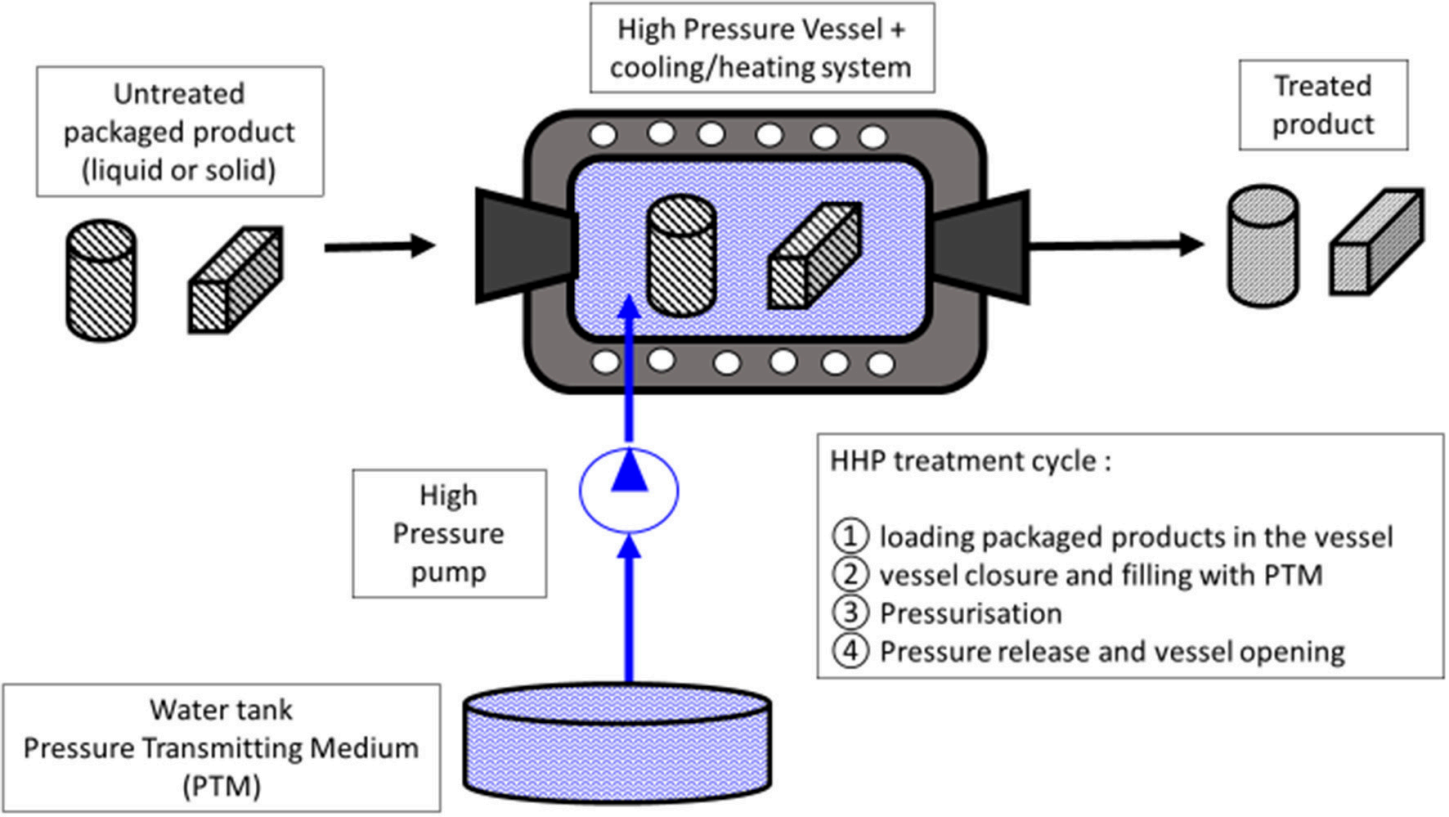
Schematic layout for a High Hydrostatic Pressure (HHP) treatment pilot.

Treatments by HHP consist in placing the product (liquid or solid) in a pressure vessel filled with the pressure-transferring medium (PTM) (generally water in food applications) that is compressed by a pump (Figure 1). Based on the Pascal or isostatic principle, the hydrostatic pressure is transmitted uniformly and immediately to the sample through the PTM. One of the major advantages of this technology, compared to heat treatments, is that the effects of pressure are not dependent on the size and geometry of the products. However, the classical limitation of heat transfer has to be taken into account. The adiabatic heat of compression is reversible and estimated to about 3°C per 100 MPa for most of foods and can reach 8–9°C/100 MPa for high-fat products (15). Hence, after pressure release, the product will return to its initial temperature (15). The three processing parameters characterizing an HHP treatment are the temperature, the pressure, and the exposure time. Generally, in the food preservation area, pressure levels between 100 and 800 MPa are applied, at mild a temperature (4–20°C), from several seconds up to several minutes (16). In the food industry, the treatment vessels typically have an internal volume ranging from 50 to 525 L. The efficacy of high pressure (HP) on biological systems is governed by Le Chatelier's principle, which provides that pressure will favor any phenomena (reaction, transition…) accompanied by a reduction in volume and will inhibit those associated with a volume increase. Due to the low compressibility of covalent bonds as compared to weak energy bonds, low molecular weight molecules, such as aroma compounds, vitamins, and minerals are rarely impacted by HP, while macromolecules, such as proteins and starch, can change their native structure (16). Historically HHP treatments have been mainly applied to food preservation, but new promising applications in food or biotechnology areas have been studied the last several last years (17–19). Barba et al. (17) present a review of the potentialities of new HHP applications, which include: (a) recover health-related compounds, (b) improve health attributes of foods through increased bioavailability of micronutrients and phytochemicals, (c) reduce allergenic potential, (d) preserve healthy lipids, (e) reduce salt intake by increased saltiness perception, and (f) reduce formation of processing contaminants (17).

### High Pressure Homogenisation

**Figure 2:**
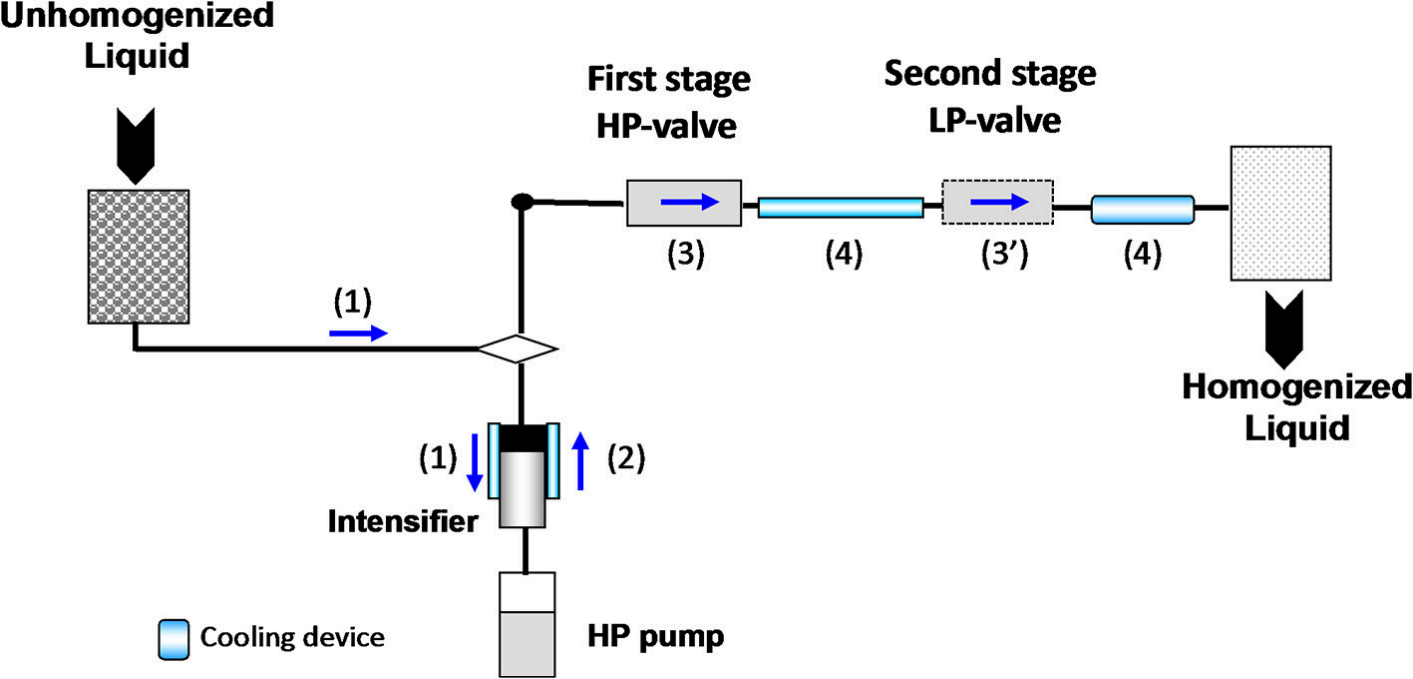
Schematic layout for ultra-high-pressure homogenization (HPH) pilot. Unhomogenized liquid is pumped to the high-pressure intensifier (1), pressurized (2) and forced through high pressure (HP) then low-pressure (LP) valves (3–3′). Fluid temperature heating is limited by cooling devices (4).

For the last decade there has been a growing interest focused on the development of dynamic high-pressure processing, i.e., (ultra)-high pressure homogenization, in food processing. Various applications of this novel non-thermal processing technology have been specifically designed with a sustainable development perspective in mind. This eco-friendly continuous processing method is liquid based, and generates a combination of physical, hydrodynamic, and thermal effects allowing for the extraction of natural compounds from suspended particles, the non-thermal stabilization of liquid foods, such as milk or juice, but also the production of extremely stable submicron emulsions (20, 21). Dynamic high-pressure processing consists of pressurizing the liquid to treat for ~10 s using a high-pressure generator, and then the fluid is forced through a high-pressure valve characterized by a very small orifice up to 2 μm (Figure 2). According to the nominal pressure level, the process is deemed High pressure homogenisation (HPH) (100–200 MPa) or UHPH (300–400 MPa). When the pressurized fluid is forced through the HP valve, the pressure drops from high pressure down to atmospheric pressure, thus inducing a significant increase of the fluid velocity. This is associated with intense shear rates. At the same time, the kinetic energy is partially converted into heat, inducing a short-life heating phenomenon (~ < 1 s). Dynamic high pressure is recognized as a non-thermal or mild thermal processing since the temperature increase is limited in the amplitude and can be easily controlled by a cooling system located just after the HP valve. After the valve-gap, the fluid flows in the chamber where turbulences, cavitation, and impacts between particles and against walls occur, as well as recirculation. At the present time, UHPH has potential as a more sustainable food processing operations, as defined by Chemat et al., and has been demonstrated at pilot scale, but upscaling to transfer the technology to industrial processing lines is just being implemented now (11). A large number of studies have been carried out to demonstrate the advantage of UHPH compared to classical processes in terms of process effectiveness, but very few studies have been dedicated to quantify the environmental impacts of UHPH (22).

### Carbon Dioxide Processes

**Figure 3:**
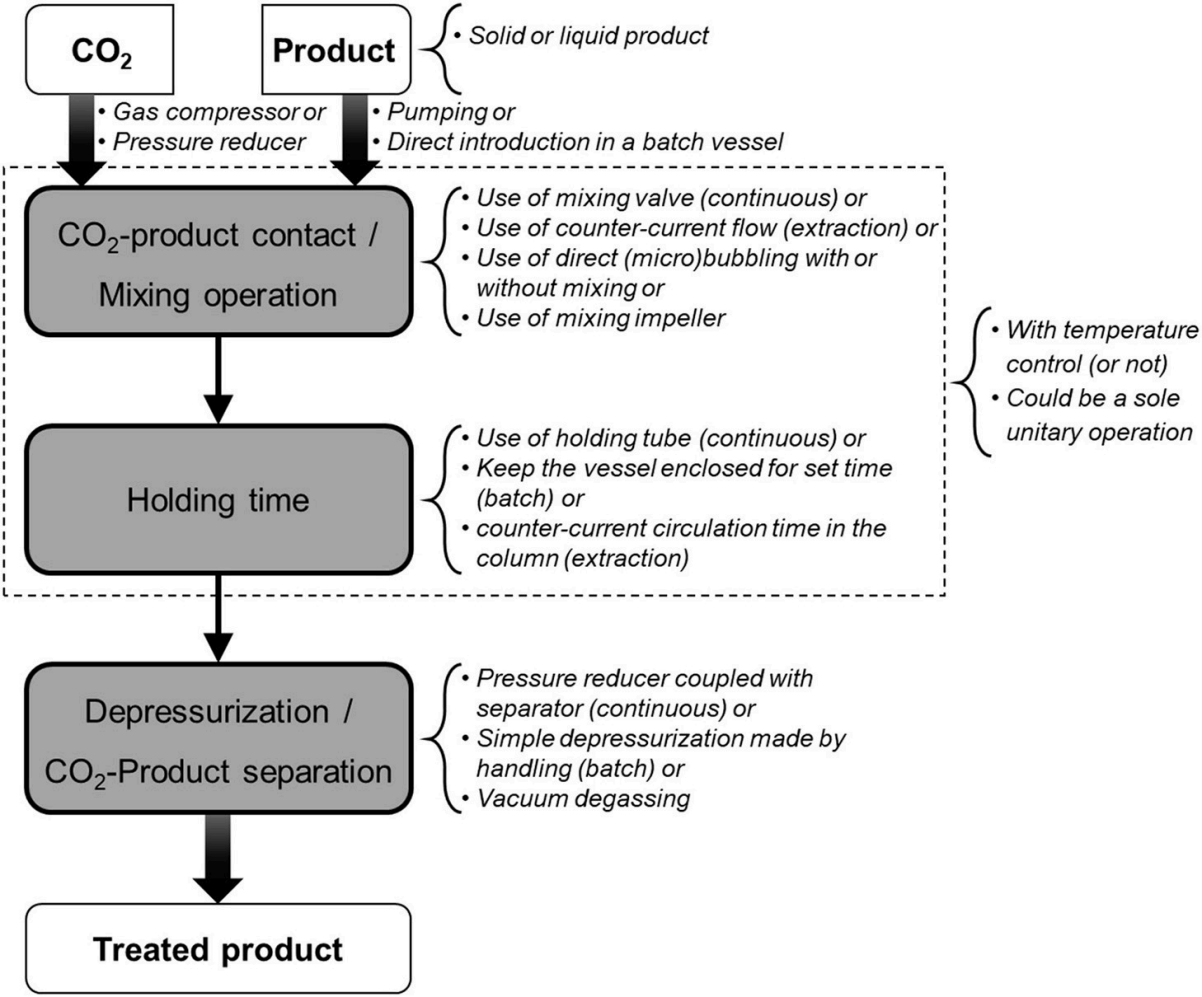
Steps and technical characteristics of CO_2_ treatments reported in Table 1.

**Figure 4:**
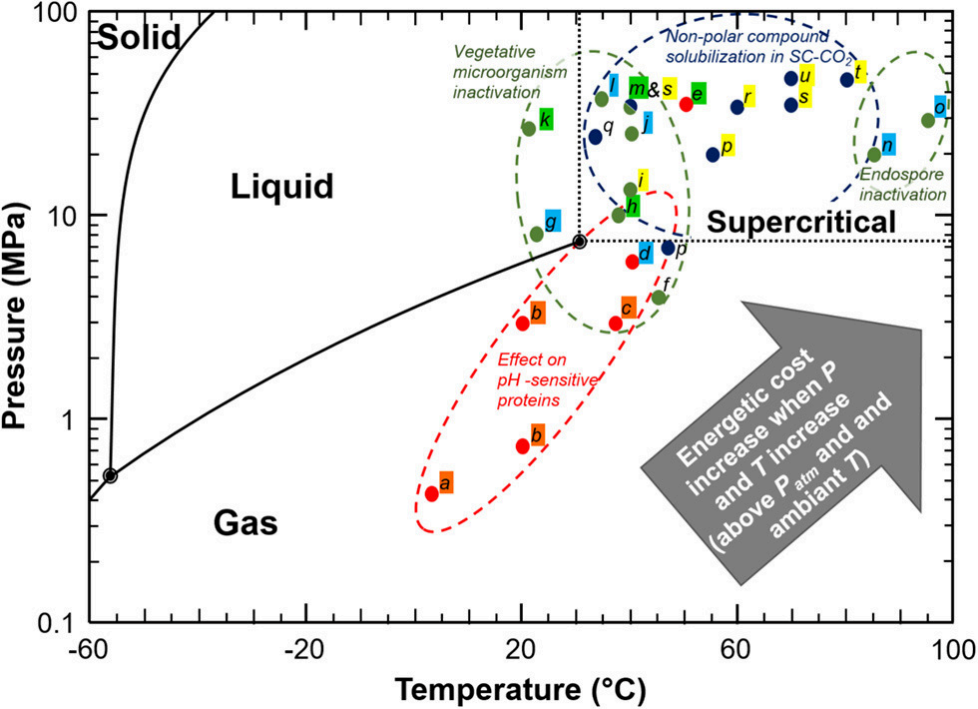
Representation of effective CO_2_ treatments on a phase CO_2_ diagram. Lettering is described in Table 1. Coloured highlight indicates the name of the different CO_2_ treatments as mentioned in the corresponding studies: Orange, CO_2_ acidification (carbonation); Blue, HPCD; Green, DPCD; Yellow, SC-CO_2_.

Carbon dioxide is a cheap, non-toxic and non-flammable compound which has been intensively studied in food processing for the purpose of pasteurization and sterilization, product texturing and functionalization, as well as for fractionation and extraction of compounds (23–26). These processes generally consist in mixing carbon dioxide under gas, liquid or supercritical state into a liquid or solid matter and maintaining a pressure/temperature condition during a sufficient period to allow for matter transfer between both (Figure 3). Among processes involving CO_2_, the most common are CO_2_ acidification (or carbonation), Dense Phase Carbon Dioxide (DPCD), High Pressure Carbon Dioxide (HPCD), and Super Critical Carbon Dioxide treatment (SC-CO_2_). Figure 4 and associated Table 1 illustrate a selection of effective pressure and temperature treatments related to these goals (27–47). Treatments, such as DPCD, HPCD and SC-CO_2_ often have overlapping pressure/temperature ranges (e.g., DPCD and HPCD can be used for CO_2_ under liquid phase). Moreover, similar technologies and applications can be identified by one or more of these names. Hence, four domains are often distinguished according to the targeted effect of the treatment (Figure 4): destabilization of the native structure of pH-sensitive proteins, inactivation of vegetative microorganisms, solubilisation of non-polar (or low polar) compounds in SC-CO_2_, and inactivation of endospores. As only few data can be found on the cost of these treatments (26), as a first approximation it can be assumed that the energetic cost increases when temperature and pressure increase. Nonetheless, for the range of applications considered (stabilization of a product, extraction, etc.), processes using CO_2_ are considered green processes, as they represent an alternative to thermal treatment, high pressure (above 200 MPa) treatment, and solvent extraction (25, 26). These processes do not generate CO_2_ but use existing resources, which is an important point for sustainability requirements (26).

**Table 1:** details about the CO_2_ processes described in Figure 4.

**Label**	**Application**		**Process name in the original paper**	**Raw material considered**	**Parameters *P* (MPa) / *T* (°C) / *t* (min)/Other**	**Targeted effect and result**		**Source**
*a*	Effect on proteins aiming at	•[-]Functionalize	Carbonation	Milk	0.32/4/45	Vectorization of a casein fraction		(27)
*b*		•[-]Fractionate/extract	CO_2_ acidification	Soy flour proteins dissolved at pH9 ($\frac{1}{2}$ diluted)	~0.7/20/~20	Soy proteins aggregation and separation	11S fractio	(28)
					~3/20/~20		7S fraction
*c*		•[-]Extract	CO_2_ acidification	Sponge	3/37/13.5	Collagen and gelatine solubilisation	12.4% yield	(29)
*d*		•[-]Texturize	HPCD	Water solution/silk	6/40/120	Silk protein gelation		(30)
*e*			DPCD	Myosin	20/50/ < 1	Myosin gelation		(31)
*f*	Pasteurization		Low-pressure CO_2_ microbubbles	Milk	4/45/5	*E.coli*	3.0 Log_10_	(32)
*g*			HPCD	Fresh cut carrot	8/22/10	Total coliform	5.0 Log_10_	(33)
*h*			DPCD	Carrot juice	10/37/45	*S. typhimurium*	3.3 Log_10_	(34)
*i*			SC-CO_2_ pasteurization	Fresh cut coconut	12/40/15	Vegetative bacteria and yeasts	2–3 Log_10_	(35)
*j*			HPCD	Milk	25/40/50	Yeast population	3.0 Log_10_	(36)
*k*			DPCD	beer	26.5/21/4.77	Yeast population	7.4 Log_10_	(37)
*l*	Pasteurization and enzyme		HPCD	Orange juice	38/34/10	*S. typhimurium*/*L. monocytogenes*	>6.1/>6.4 Log_10_	(38)
*m*	inactivation		DPCD	Red grapefruit juice	34.5/40/7	Total aerobic cultivable bacteria	5.1 Log_10_	(39)
*n*	Sterilization		HPCD	Bacterial suspension	20/85/30	*B. subtilis* spores	1.3 Log_10_	(40)
*o*			HPCD	Bacterial suspension	30/95/120	*B. stearothermophilus* spores	5 Log_10_	(41)
*p*	Non-polar (or low polar) compound		CO_2_ expanded-etOH	*Haematococcus pluvialis*	7/45/120 /~50% eth.	Astaxantin extraction	124% yield	(42)
	extraction		SC-CO_2_ extraction		20/55/120		2.5% yield
*q*			SC-CO_2_ + co-solvent	Plant coproduct	25/33/60/~50% eth.	Terpene and resin	9% yield	(43)
*r*			SC-CO_2_ extraction	Plant coproduct	34.5/60/30	Terpene and resin	5% yield	(44)
*s*			SC-CO_2_ extraction	*Spinacia oleracea* L.	35/70/360	Carotenoid	Max yield	(45)
					35/40/360	Polyphenol	Max bio-activity
*t*			SC-CO_2_ extraction	Dehulled palm kernel	48.3/80/40	Palm kernel oil	49 g/100 g	(46)
*u*			SC-CO_2_ extraction	*Euterpe oleracea*	49/70/30	Berry oil	45.37% yield	(47)

### Pulsed Electric Fields

**Figure 5:**
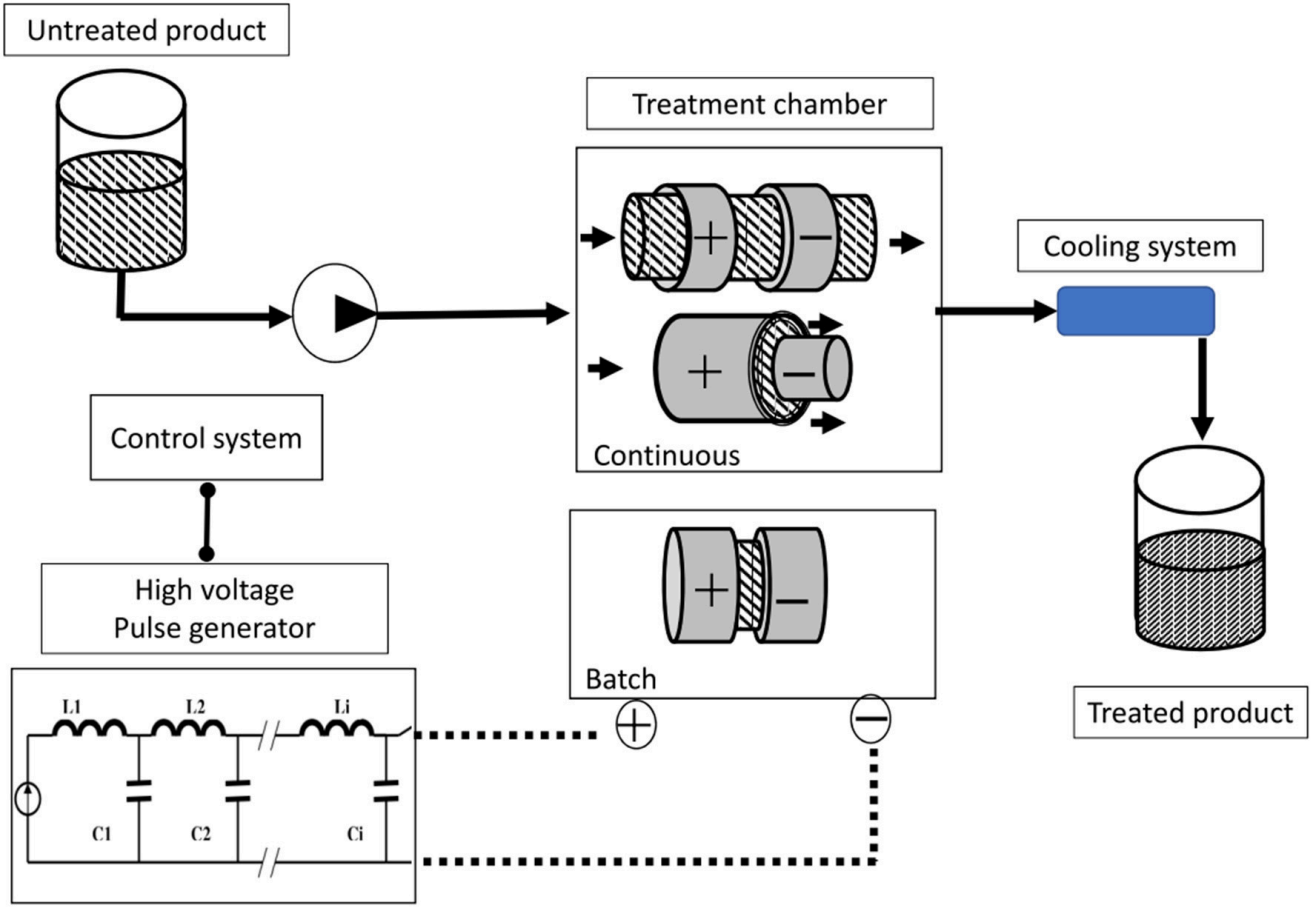
Schematic layout for a Pulsed Electric Fields (PEF) treatment pilot [adapted from Picart and Cheftel (48)].

PEF is based on the application of short electric pulses (usually 1–20 μs, but with a range of 50 ns to several milliseconds) with a high field strength (15–80 kV·cm^−1^) to samples placed between two electrodes. This can be done in a batch or continuous treatment chamber (Figure 5). PEF is considered a non-thermal processing method as well. If a biological cell is exposed to a sufficiently high electric field, its membrane becomes permeable to molecules that otherwise cannot pass through; this behavior is referred to as “electro-permeabilization” or “electroporation” (49–51). Depending upon process parameters (essentially electric field strength and pulse duration) and total energy input, PEF applications can be divided into different types: microbial inactivation (15–40 kV/cm to 1,000 kJ/kg); sludge disintegration (10–20 kV/cm to 50–200 kJ/kg); improvement of mass transfer in plant or animal cells (0.7–3.0 kV/cm to 1–20 kJ/kg); reversible electro-permeabilization of biological cells for DNA transfer (0.7 kV/cm to 1–10 kJ/kg); induction of stress response (0.5–1.5 kV/cm to 0.5–5 kJ/kg) (15). Examples of applications and experimental conditions of PEF for different food processing techniques have recently been reviewed by Barba et al. (52) and Chemat et al. (11).

### Membrane Processes

Membrane separation processes are also good candidates to achieve more sustainable food processing operations. Indeed, membrane systems can be operated in continuous mode, which limits start up and shut down as well as cleaning procedures and leads to consistent product quality. Working temperatures are low (i.e., ambient or temperatures < 0–80°C) without involving phase changes or chemical additives; the qualities of heat-sensitive products are thus preserved, so waste generation as well as energy costs are limited. Furthermore, they can be used to achieve various objectives of separation (i.e., concentration/clarification, purification, fractionation, and extraction) but can also permit the recovery and reuse of by-products for a more effective utilization of raw materials. Finally, they are modular and thus easy to scale up. All these advantages make them one of the most promising technologies in terms of sustainable processes (53, 54).

**Figure 6:**
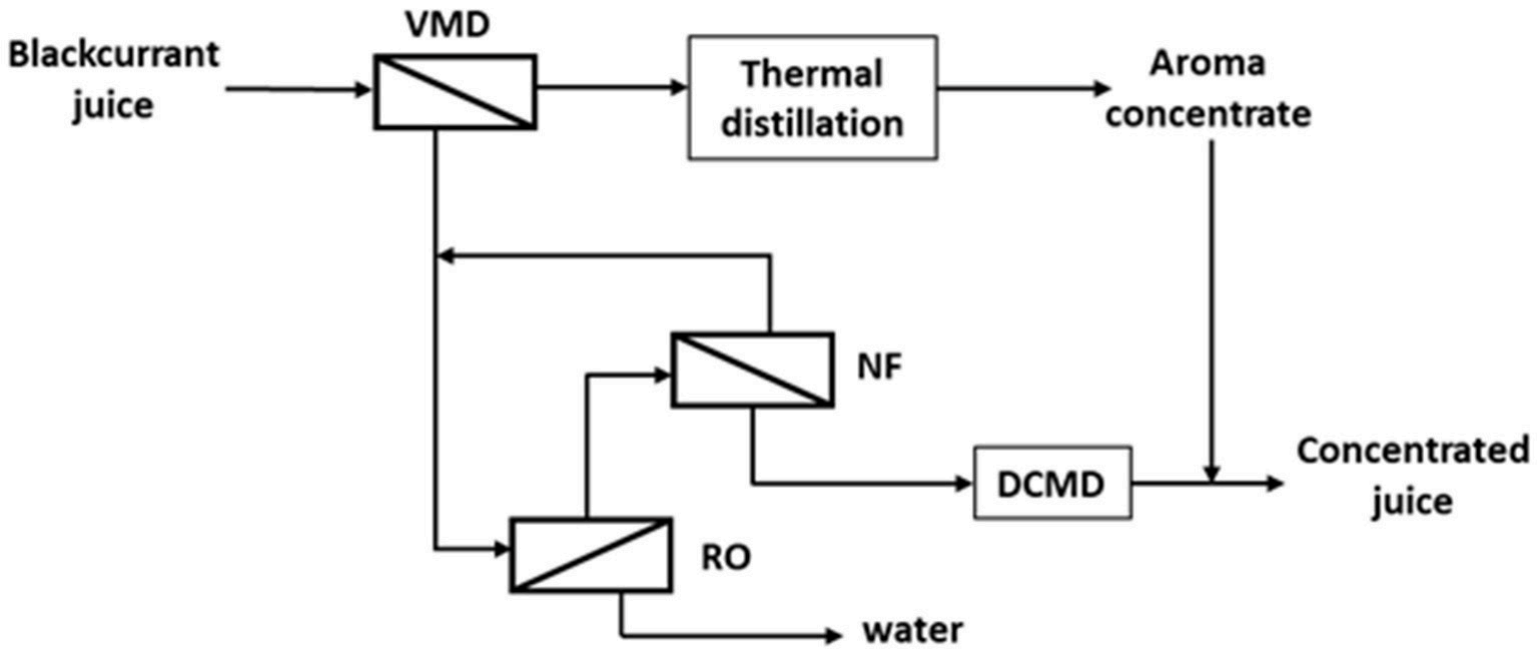
Schematic process layout for concentration of blackcurrant juice based on membrane processes (VMD, vacuum membrane distillation; RO, reverse osmosis; NF, nanofiltration; DCMD, direct contact membrane distillation) adapted from Sotoft et al. (55, 56).

At the heart of all membrane processes, the membrane acts as a thin selective barrier or interface between two phases, and which ensures the transfer of solvent and/or solutes due to a driving force (Figure 6) (i.e., pressure gradient in microfiltration (MF), ultrafiltration (UF), nanofiltration (NF) and reverse osmosis (RO), concentration gradient in pervaporation (PV) and membrane contactors (MC), electrical potential gradient in electrodialysis (ED), or temperature gradient in membrane distillation (MD). According to the specific membrane properties (e.g., nature (organic or inorganic) structure (dense or porous), etc.) the separations result from different mechanisms (sieving, charge, or diffusion effects) allowing different processing objectives to be achieved (57).

Of course, other processes are also considered potential candidates for green technologies (e.g., ultrasound, microwave, Instant Controlled Pressure-Drop, etc.). However, this review paper is limited to establishing an inventory of the benefits and energetic treatment costs for the five aforementioned processes with respect to food stabilization, extraction, and water recycling.

## Non-Thermal Stabilization

The stability of food products over time is certainly one of the most important considerations of quality for food manufacturers. To ensure this stability, food manufacturers have to target different goals such as:

- limiting microbial growth in food during storage by direct inactivation of microorganisms, or by generating conditions limiting their growth (such as acidification),
- maintaining (or increasing and then maintaining) the nutritional quality of the raw material by limiting/controlling foodstuff denaturation during processing, and then limiting enzymatic alteration (the most often by direct inactivation during the process) or oxidation (by limiting formation of reactive oxygen species),
- avoiding any dephasing or exudate release by appropriate texturization.

The five alternative processes reviewed in this paper have demonstrated their effectiveness to stabilize various food stuffs by achieving the objectives of stability described above. Indeed, many papers show that these alternate processes can result in comparable or even better stability than traditional thermal processes, but with comparable or lower energetic costs.

### Hydrostatic High Pressure

High hydrostatic pressure has been applied to food preservation since 1980–1990. It is widely reported that HHP treatments above 350 MPa permit the inactivation of spoilage microorganisms and enzymes at low temperatures, and can also induce the denaturation of macromolecules, such as proteins, lipids, and starch (18, 58–60). Cell membranes are often considered the first site of injury in pressure-inactivated microorganisms. Even if the exact inactivation mechanisms are not yet fully understood, experimental data show that HHP affects cell membrane permeability and cellular structures, probably through alteration of proteins and lipids (19). As observed for other preservation techniques, differences in the resistance of microorganisms to HHP have also been reported: yeasts and molds are less resistant than vegetative bacteria cells; Gram+ bacteria are generally more resistant than Gram- bacteria; bacterial spores are highly resistant to HHP conditions.

Currently, HHP processing is a well-established non-thermal technology for increasing food safety and/or extending the shelf-life of refrigerated foods of high value (61). Products pasteurized by HHP processing show nutritional and organoleptic properties similar to those of the raw/fresh products, which is contrary to conventional thermal treatments (62, 63). Comparative studies on non-thermal preservation techniques reported that the effects of HHP on microbiological quality and physicochemical properties of juices were higher, or similar to, those observed with other non-thermal techniques, such as PEF (62). However, contrary to PEF, HHP processing is increasingly used at commercial, industrial scale for specific market niches, such as vegetable products (~33%), meat products (~30%), juices and beverages (~12%), seafood, and fish (~15%) (13, 16, 61). In 2011, there were 125 HPP units installed in over 60 companies, producing 250 different HHP-treated products. An HHP industrial unit (600 MPa, room temperature, overall production around 5,000 kg/h) is almost as expensive as a PEF industrial unit, and costs between 0.77 and 3.15 million US $, depending on the vessel volume (55–420 L). Currently, the main suppliers of HHP installations are Avure technologies (USA), Hiperbaric (Spain), HPBioTech (France), Multivac (Germany), ThyssenKrupp (Germany), and Steribar HPP (Hydrolock, France). In terms of production costs, high-pressure processing requires power to increase pressure. Due the adiabatic heat of compression, the theorical total energy input for processing pure water at 600 MPa is about 122 kJ/kg. The cost of HHP will also depend on the total cycle time (i.e., pressure increase, holding, and loading/unloading times), vessel filling ratio, energy, labor, and capital costs (61). Recently, Sampedro et al. estimated that the total cost of orange juice pasteurization using HHP (0.107 $/L) was 7-fold higher than that of conventional thermal processing (0.015 $/L), and 3-fold higher than that of PEF treatment (0.037 $/L) (64). They also estimated that non-thermal processing technologies may have higher environmental impacts (in terms of CO_2_ production) than traditional thermal pasteurization. Aganovic et al. discussed similar trends for the pasteurization of tomato or watermelon juice (65). They calculated that the specific energy uptake was about 0.20, 0.12, or 0.04 kWh/L of juice by using HHP, PEF, or thermal treatment, respectively. From the environmental (Life Cycle Assessment) perspective, no great differences in environmental impacts have been observed over the three investigated technologies with “gate to gate” system boundaries. A slightly higher impact was observed for HPP, followed by PEF and thermal treatments. Even though the differences of processing stage were assigned to the use of energy, the largest environmental impact was associated with 250 ml PET bottles production (~85%) (65).

Finally, it is noteworthy that inactivation of bacterial spores can be obtained with HHP by following different strategies of which: (1) full inactivation in one step by severe temperature or pressure-temperature combinations (up to 800 MPa and 75°C), or (2) germinating spores by temperature and/or pressure (50–300 MPa) and then inactivating them in a subsequent temperature or pressure/temperature treatment (>400 MPa) (16, 19). From an energetic point of view, combining thermal and high-pressure sterilization allows for an overall reduction of energy input of 20%, as compared to thermal sterilization alone, due to the recovery of adiabatic heat of compression (15).

### High Pressure Homogenisation

High pressure homogenisation (HPH) was initially dedicated to cell disruption and removal of intracellular compounds from microorganisms (66). A growing interest for over 15 years was focused on the mild non-thermal stabilization of food; UHPH has been evaluated as an alternative to conventional heat treatments of liquid foods to inactivate microorganisms and endogenous enzymes, and consequently increase the shelf life of products (20, 21, 67, 68). The efficiency of UHPH for microbial inactivation of spoilage microorganisms and foodborne pathogens has been demonstrated to increase with the pressure level, the extent of recycling through the homogenizer, the temperature of the processed fluid, but depends on the characteristics of the food matrix (e.g., water and fat contents, viscosity, pH, etc.) and also the type of microorganisms present.

Several studies have demonstrated that HPH is equivalent to food pasteurization. HP technology developments have been many, especially concerning HP intensifiers, materials resistant to HP, and also sophisticated homogenization valves with seats and needles built in ceramic or coated with artificial diamond have allowed for increases in nominal pressure levels up to 350–400 MPa (20), and thus the application of UHPH for bacterial spore inactivation (68–70). However, UHPH has to be combined with high inlet temperatures for liquid foods to obtain a treatment equivalent to thermal sterilization (71, 72). At the present time, the vast majority of studies have focused on the quantification of the UHPH inactivation efficiency, and on understanding of the impacts of the various phenomena involved in this process on the inactivation of microorganisms or enzymes. Even though it has been determined that this process can pasteurized liquid foods while preserving sensorial and nutritional qualities, and can limit the use of high temperatures, very few studies have examined or evaluated the sustainability aspects of UHPH processing (21, 73). UHPH was recently compared to classical thermal processing by Valsasina et al. milk production (22). They compared the environmental impacts of UHPH technology to those of a conventional thermal treatment (UHTH—ultra-high temperature treatment and homogenization) using life cycle assessment (LCA). At a pilot scale, a lower energy consumption was determined for UHPH compared to UHTH, with consequently a significantly lower carbon footprint for UHPH. Electricity production was evaluated as the main input in the LCA for UHPH and UHTH. By introducing energy recovery in the UHPH equipment, a significant improvement of the LCA score for UHPH could be obtained. UHPH being available currently only at a pilot scale, the evaluation of LCA at industrial scale has to be carried out by upscaling approaches (22). The main drawback concerning the potential of UHPH to stabilize liquid foods at industrial scales concerns equipment development (e.g., pumping, intensifiers, and valves) that must ensure the flow capacity of production lines. This is a challenge for the equipment manufacturers. Currently, piston-gap homogenizers are proposed by Avestin (Canada), APV (UK), Bee International (USA), GEA Niro Soavi (Italy), Stansted Fluid Power (UK), and Ypsicon (Spain) and another system called microfluidizer is marketed by Microfluidics (USA).

### Carbon Dioxide Processes

CO_2_ processes have been investigated for their ability to stabilize products according to several criteria: bacterial content (26), undesired enzymatic activity (39), texture (31), or nutritional quality (27, 35, 39). To our knowledge, no experimental data have been published on the cost of processes using CO_2_ for these purposes (26). Nonetheless, the treatment intensity in terms of pressure/temperature can be discussed and compared with currently used technologies. Under pressure, CO_2_ dissolves in aqueous media by forming carbonic acid, which lowers the pH (74). For pH-sensitive proteins, mild treatments involving pressures between 0.1 and 7 MPa and temperatures between 4 and 45°C for < 1 h can be sufficient to inactivate polyphenol oxidase in various food products (74), form stable gels with silk proteins (30), and stabilize curcumin in casein micelles (27). These mild process parameters can be considered eco-efficient compared to conventional treatments (temperatures above 55°C or addition of chemicals) required to obtain equivalent protein modifications (30, 74). The inactivation of vegetative microorganisms by CO_2_ requires stronger conditions, and has been demonstrated to be the consequence of simultaneous effects: lowering of pH and CO_2_ bubble formation when pressure is released can induce chemical alterations of structural proteins and enzymes, as well as mechanical disruption of cell membranes, respectively (26, 74). Inactivation of vegetative microorganisms by CO_2_ (sometimes called CO_2_ pasteurization) has been intensively studied for temperatures ranging between 20 and 50°C and pressures ranging between 4 and 60 MPa; this technique represents another alternative to thermal pasteurization (which is most often over 80°C) and to high pressure inactivation (over 150 MPa) (26, 74). Effective inactivation of more than 3 Log_10_ reductions for various microorganisms have been obtained after a few minutes to < 1 h of treatment (Table 1). For products with substantial protein content, like milk or peach juice, undesirable texture modifications have been reported due to protein aggregation (32, 75). However, for many products with low suspended protein content, e.g., apple and grapefruit juice, beer, sake, or solid fresh-cut vegetables, CO_2_ pasteurization appears to result in better quality (texture, color, flavour, and nutritional properties) than equivalent thermally treated products (26, 32, 35, 39). Several patented systems exist, and industrial equipment have already been available for more than a decade from manufacturers like Praxair or GEA groups, but industrial applications are still in development. In fact, as reported by Spilimbergo et al. the main drawback remains the equipment cost compared (for example) to heat exchangers. In their paper, they briefly present an estimation of the cost of pasteurization of apple juice by CO_2_ treatment as compared to the thermal pasteurization using tubular heat exchangers. They claim a treatment cost of only a few cents per liter higher for CO_2_ treatment compared to thermal treatment, and they highlighted that larger scale units can be economically feasible (26). Based on current scientific knowledge, there are only few patents available, and no currently used equipment include a CO_2_ recycling unit. Finally, CO_2_ sterilization, i.e., the inactivation of endospores, has been less documented than inactivation vegetative microorganisms. It has been reported that effective CO_2_ treatment involves temperatures above 85°C (40, 41) which necessarily consumes more energy than the CO_2_ pasteurization unit. Rao et al. (40) indicated that the pressure level (below 30 MPa) has fewer effects than increasing temperature, but the fact is that supercritical CO_2_ allows for endospore inactivation at temperatures for which temperature alone has no effect. In fact, endospores are known to be extremely resistant organisms which required temperatures above 120°C in food processes to be inactivated (76). Therefore, CO_2_ sterilization can be a promising process, but require further investigations.

### Pulsed Electric Fields

The potentialities of PEF as an alternative technique to thermal pasteurization has been widely investigated in the last six decades. A large number of studies have shown the efficacy of PEF treatment for the inactivation of most spoilage and pathogenic microorganisms. The main factors governing microbial inactivation by PEF are the treatment parameters (i.e., electric field strength, total treatment time, energy density input, pulse width and shape, and pulse repeat frequency), the microbial characteristics, and the product parameters (electrical conductivity and pH) (48, 77). Even though exact correlations between process parameters and microbial inactivation are not yet fully elucidated, it has been extensively demonstrated that (1) increasing the electric field strength and/or the total treatment time increased microbial inactivation; (2) the electric field strength applied must exceed a critical value that ranges from 5 to 15 kV/cm, depending upon the microorganism or treatment chamber. Differences in the resistance of microorganisms to PEF have been widely reported: vegetative bacterial cells are more resistant than yeasts or molds; Gram+ bacteria are generally more resistant than Gram- due to the differences in cell wall membranes; and bacterial spores generally resist electric pulse conditions which inactivate vegetative cells (48, 77). The potential of PEF to achieve sufficient reduction for most of the spoilage and pathogenic microorganisms has been proven in a broad variety of liquid foods, including fruit and vegetable juices, model beer, milk, and liquid egg (52, 62). Even if there are still conflicting views about the inactivation of enzymes by PEF, data available show clearly that plant enzymes are much more resistant to PEF than vegetative microbial cells (78). PEF treatments have been reported to induce inactivation of enzymes involved in plant spoilage such has pectin methyl esterase, and peroxidase as PME (up to 97%), polygalacturonase (up to 76.5%), polyphenol oxidase (up to 97%), peroxidase (up to 97%), and lipoxygenase (up to 64%). However, to reach these levels of enzyme inactivation large specific energies (1,066–44,000 kJ/L) are required, while only 50–1,000 kJ/kg are sufficient for microbial inactivation (78). More recently, numerous studies have reported that PEF-pasteurization of plant-based beverages at mild temperatures can minimize changes in their physicochemical characteristics (e.g., pH, color, aroma, and flavor) and can significantly improve the nutritional properties of beverages due to retention of higher amounts of health-related biomolecules (e.g., vitamins, polyphenols, carotenoids…), as compared to thermal processing (52, 79–81). Despite the large quantity of scientific data available in the literature about the benefits of PEF processing and various technology transfer projects, there is still very little industrial implementation of PEF for pasteurization of liquids, and it is primarily limited to waste water treatment (82).

PEF treatment is usually considered to have a high initial investment costs and elevated processing costs, with higher energy input than a thermal process with heat recovery capacity (83–85). For applications requiring high electric field strength and/or high energy input (20–40 kV/cm, 100–1,000 kJ/kg), the investment costs is estimated to be in the range of 2–3 million US $ for an industrial scale device of 5 t/h (15). Recently, Sampedro et al. estimated that the total cost of orange juice pasteurization using PEF was about 0.037$/L of juice, against 0.015 $/L for thermal pasteurization, of which capital costs accounted for 54% while utility charges (mainly electricity) accounted for 11% (85). Aganovic et al. calculated that the specific energy uptake to pasteurize tomato or watermelon juice was about 0.12 or 0.04 kWh/L of juice by using PEF or thermal treatment, respectively (65). From an energetic point of view, improvement of PEF processing (higher microbial and enzyme inactivation with lower field strength and less electrical energy) can be achieved by combining PEF with other treatments (such as pH, antimicrobials or heat) (11, 62). In particular, using elevated inlet temperatures (up to 55°C) allows the reduction of energy input to achieve the same inactivation level, and the need to preheat the product provides a potential to recover the electrical energy dissipated into the product (15). However, it would be important to optimize the process conditions while still maintaining the potential quality benefit of PEF (62, 65). In the last few decades, the application of PEF has also been studied as pre-treatment for other food preservation techniques. The main studies dealing with PEF-assisted drying or freezing have recently been reviewed by Barba et al. (52) and Chemat et al. (11).

### Membrane Processes

Initiated in the early 1960s, the industrial interest in membranes firstly focused on water desalination. But membranes were rapidly introduced in food processing since they permit the replacement of conventional thermal technologies or reduce their negative impact during stabilization processes. For instance, microfiltration (MF) is currently widely used for cold stabilization of milk prior to transformation into cheese or to extend its shelf life without applying a time-temperature treatment. More than 99% of the bacterial are removed without thermal degradation of protein or off-flavor development. Concentration, polarization, and membrane fouling—the main drawbacks of membrane filtration, can be limited by applying a uniform transmembrane pressure (TMP) all along the membrane via the circulation of the permeate co-current with the retentate (for example, Bactocatch system®, AlphaLaval; Invesys® APV). It is also possible to use ceramic membranes with a linear hydraulic resistance gradient (e.g., GP Membralox® membrane Pall-Exekia; Isoflux® membranes Tami-Industries) (86). Another important application of membrane technologies in dairy processing is the MMV procedure, patented by Maubois Mocquot and Vassal in 1969, in order to pre-concentrate milk by ultrafiltration (UF) before cheese making (87). The MMV process offers many advantages (i.e., higher overall cheese yield compared to traditional processing, conversion of cheese production to a continuous operation, and elimination of the need for large storage tanks) leading to high operational efficiency with lower overall capital costs (88).

The first applications of membranes in the dairy industry were mainly driven by product innovation. However, the combination of membrane technologies with classical thermal operations or with other emerging technologies can significantly reduce the energy consumption during milk powder production, which is responsible for about 15% of the total energy used in the dairy industry. Indeed, huge amounts of energy are required for evaporation and spray drying steps. Preconcentrating the milk by reverse osmosis (RO) before thermal evaporation is advantageous because the low energy consumption compared to evaporation (14–36 kJ/kg water removed, and 300 kJ/kg water removed, respectively) (89). Nevertheless, according to Moejes and van Boxtel, the best option would be to replace heat evaporation by a combination of a radio frequency heating system which generates heat directly within the product by using electromagnetic waves combined with membrane distillation (MD), which is a membrane contactor process (89).

In contrast to reverse osmosis, which is a pressure-gradient process involving a dense membrane, membrane distillation involves a porous hydrophobic membrane in order to create an interface for mass transfer between the solution to be concentrated (feed) and the stripping solution (water at low temperature) (i.e., permeate). The hydrophobic nature of the membrane prevents the penetration of the liquid phase and creates a liquid-vapor interface at the entrance of each pore. Due to the temperature gradient, evaporation occurs at the feed side; then the volatile compounds (mainly water in the case of milk concentration) diffuse across the membrane and are condensed into a liquid phase (Direct Contact Membrane Distillation (DCMD) or over a cool surface (Air Gap Membrane Distillation (AGMD) or are removed by a gas flow [Vacuum Membrane Distillation (VMD) and Sweep Gas Membrane Distillation (SGMD)] (90). In contrast to reverse osmosis, MD presents a low flux sensitivity toward concentration of the processed fluid and thus permits a high concentration level. Nevertheless, the implementation of MD for milk production requires further investigation since the process efficiency is still limited by membrane fouling (55).

Membranes technologies also present high potentialities in beverage processing (91). MF and UF can advantageously replace conventional filtration processes for clarification and microbial stabilization of juice (i.e., high product qualities, continuous processing, low waste, etc.); juice concentration can be carried out by RO up to a limited concentration rate (up to 30% TSS (total soluble solute), but higher TSS (up to 60%) can be achieved by osmotic membrane distillation (OD). In this process a porous hydrophobic membrane is used to separate juice from a stripping solution with a low water activity (i.e., a brine). On the contrary to MD, the driving force is no longer the temperature gradient, but the water vapor pressure gradient induced by the difference in the water activity of the aqueous solutions which flow along the membrane. Therefore, it is not necessary to heat the solution to be concentrated; its qualities are thus preserved. However, corrosion and high production costs due to the use of concentrated brine can be challenges to using OD. To avoid these drawbacks, Sotoft et al. proposed an alternative process for blackcurrant juice concentration (56). This new process combines different membrane techniques, allowing the production of aroma concentrate and concentrated juice. Firstly, the filtered blackcurrant juice is treated by VMD. The permeate, which contains aroma, is further concentrated by distillation while the retentate is then concentrated by a combination of membrane processes (RO, NF, and DMC). Finally, aroma extract is added to the concentrate (Figure 6).

## Eco-Friendly Extraction And Valorization Of Bioresources

Valorization of bioresources mainly consists in recovering high-value compounds from raw materials, by-products, or food wastewater. Extraction of intracellular molecules often involve cell damage techniques, such as fine mechanical fragmentation, thermal, chemical, and enzymatic treatments. These conventional techniques often require a significant amount of mechanical or thermal energy, long time steps, use of toxic solvents, or high temperatures that can degrade thermolabile compounds, and lead to a non-selective extraction. In the last few decades, emerging non-thermal technologies developed for food stabilization have also shown promising capabilities to be useful tools for more efficient and sustainable extraction/separation processes.

### Hydrostatic High Pressure

HHP can damage cell membranes and then increase their permeability and enhance mass transfer rates of intracellular molecules (15, 19). However, HP-assisted extraction is a quite recent application of HHP processing. High hydrostatic pressure (HHP) treatment (200–700 MPa, moderate temperature) has been successfully implemented to extract bioactive compounds (phenolic compounds, carotenoids, glucosinolates…) from natural sources (grape, *Maclura pomifera* fruits, berries, tea leaves…) or by-products (grape, tomato, and citrus) (17, 52, 92–94). Diffusion of bioactive compounds could be done under pressure in water mixed or not mixed with other solvents, and effectiveness depends on pressure level, pressure holding time, liquid/solid ratio, type of solvent, and solvent concentration (17).

Overall, as compared to conventional solvent-extraction, HP-assisted extraction allows higher extraction yields to be obtained, with a shorter process time and a reduction of the use of toxic solvents. Even though only lab-scale studies have been performed on this topic until now, and without energetic and economical estimations, HP-assisted extraction represents a promising technique to improve process efficiency and thus sustainability.

### High Pressure Homogenization

A peculiar interest of dynamic high pressure concerns the eco-extraction of valuable bioactives from different types of biomass, such as algae or microbial organisms (95–97) but also from by-products or co-products to up-cycle them (98, 99). High pressure homogenisation (HPH) is mainly appropriate for these applications since the pressure level applied is more often up to 150 MPa, and there is a link between the particle gap width of HPH equipment and the particle size. The bioactives extracted are particularly prized for their biological activity such antimicrobial or antioxidant activity, but also for their nutritional properties. As with mechanical technology, HPH is used to carry out a physical disruption of organism cell membranes or to reduce particle size. In the first case, the disruption of cell membranes due to the combination of turbulence, recirculation and cavitation phenomena but also impingements on the chamber allows the non-selective release of the intracellular fluid and also cellular organelles (66). In the second type of application, also called nanosizing, HPH is applied to reduce the size particle and so to increase the exchange surface and consequently the particle activity. The efficiency of HPH technology to disrupt membrane cell is influenced positively by processing parameters: the pressure level and the number of passes (95), but strongly depends on the macrostructure of the cell wall (97). From a sustainability point of view, HPH allows the extraction of intracellular valuable components without the addition of solvents or chemicals, limiting waste to be reprocessed. By nanosizing of bioactive plant material, High pressure homogenisation (HPH) could be an alternative, less time-consuming and lower cost compared to the conventional protocol requiring extraction, fractionation and isolation (99).

Concerning bio-refinery applications, microalgae is a fast growing sector, and has a significant activity in developing operation units less costly in the downstream process where the key step is the cell disruption consisting in breaking or weakening the cell wall integrity. Early studies were carried out at low concentrated algal dispersions (< 5%, w/w DB) and have concluded that HPH energy consumption was significantly higher than the potential energy output of algal-derived biodiesel (100). Yap et al. investigated the influence of the feed concentration on the HPH capacity, power draw and cell disruption efficiency for *Nannochloropsis sp*. suspensions (0.1–25%, w/w DB) (101). HPH efficiency was independent of homogenizer feed concentration and solely dependent on the pressure level. Besides, HPH could represent between only 6% the energy content of the resulting biodiesel (conditions: (60 MPa, 25% solids, 30% TAG). Concerning the energy consumption, Safi et al. quantified the specific energy input based on the inlet pressure, the number of passes and the pump efficiency expressed per unit of treated biomass for the disruption of *Nannochloropsis gaditana*, which is microalgae characterized by a rigid cell wall, treated at a high concentration (100 g/L) (102). Additionally, the energy input was correlated to the protein yield. HPH was concurrently compared to PEF, bead milling and enzymatic treatment. HPH resulted in the lowest specific energy input related to the obtained protein yield and an energy cost per unit of released protein evaluated was between 0.15 and 0.25 *e*/kg (compared to 2–20 *e*/kg in case of PEF).

A specific drawback has to be highlighted: HPH induces total cell disruption and is consequently characterized by a poor selectivity, so downstream processing is required to achieve high purity for a specific target compound.

### Carbon Dioxide Processes

The use of pressured CO_2_ to separate and extract proteins has been investigated for various substrates like collagen/gelatin from sponge or soy flour soluble fraction at alkaline pH (28, 29). As previously described, this method is based on the control of the aqueous media pH decrease to solubilise or to aggregate protein. The precise pH control by the pressure applied allows for efficient recovery and sequential separation. The conditions required are quite low, with a pressure up to 3 MPa and 25°C. This process clearly represents an eco-efficient alternative to an equivalent chemical acidification method to separate and extract protein fractions. Nonetheless, no data on the economical aspect has been published yet.

The most common food processes using CO_2_ is supercritical CO_2_ extraction. Since the first industrial implementation in the 1970's with coffee decaffeination, this process has been progressively investigated and industrially implemented for a wide variety of food products, including solid vegetable and aqueous or oily liquid. In fact, CO_2_ has moderate critical conditions (*T* = 31°C/*P* = 7.38 MPa) and has been developed as an alternative to the energy costly and pollutant reference methods using organic/organochlorine solvents and distillation (103). SC-CO_2_ extraction typically operates for pressure ranging from 8 to 50 MPa and temperature between 40 and 80°C (Figure 1). The highest operating pressure/temperature conditions are used for lipid and other non-polar high molecular weight compounds, with *P* > 28 MPa and *T* > 60°C, whereas moderate temperature, or both temperature and pressure conditions are frequently used for other compounds like aromatic compounds, ethanol, polyphenol, and hydrophobic vitamins (11, 23, 104).

However, SC-CO_2_ extraction is not so common in industrial applications (< 40 industrial facilities have implemented in Europe in 2003), and there are still few detailed papers dealing with economic aspects (26, 105). According to Perrut (2000), the equipment cost is relatively high and increases linearly with its size. However, SC-CO_2_ extraction has a moderate exploitation cost, so finally the production cost per kilogram of fed matter can be very competitive with the conventional processes, with several advantages in term of green label (105, 106).

Several studies have investigated the potential of SC-CO_2_ extraction to reduce waste by valuing co-products, such as oil recovery from palm kernel (46) or lipid and terpenics high value compounds from bagasse left after latex extraction (43), but related economic aspects are insufficiently documented. Otherwise, recent papers have investigated the possibility to increase the number of compounds that could be recovered (including more polar compounds like fatty acid, phospholipids, certain carotenoids…) by using co-solvent extraction using 5–50% ethanol for process fluid (11, 42, 104). Most often these works have shown higher recovery yield and faster extraction for pressure and temperature closest to the critical point than for SC-CO_2_ alone. Even more recently, carbon dioxide expanded solvent extraction techniques (i.e., the process fluid used is a mixture organic solvent and CO_2_) have been developed and seem even more efficient than co-solvent extraction for the same operating conditions (11, 104, 107).

### Pulsed Electric Fields

Based on its capacity to permeabilize plant cells at moderate electric fields (0.7–3.0 kV/cm), the application of PEF has been investigated as a pretreatment to improve mass transfer of water or intracellular compounds from vegetable tissues or bio-suspension cells. A large number of studies have been devoted to this issue and showed that PEF-pretreatment represents a promising green alternative method for different applications, such as diffusion extraction, osmotic treatment, pressing extraction, drying and freezing. Besides increasing the mass transfer, PEF-assisted processing showed other advantages as compared to conventional ones, with improvement of extraction yields, decrease of processing time, decrease of process intensity (temperature, solvent…), and reduction of heat-sensitive compounds degradation (11, 52, 108, 109). The important features of moderate PEF treatment, as compared to other green alternative methods, are the possibility of pore resealing after treatment and the formation of different pore sizes in electroporated cell membrane depending on PEF conditions. Therefore, intracellular compounds of different molecular size may be selectively recovered and more easily purified under the PEF treatment (110). PEF-assisted extraction has been particularly investigated for extraction by diffusion of colorants (chorophylls, carotenoids, betalains…), sucrose, polyphenols, polysaccharides, proteins, and others secondary metabolites from vegetal, roots, mushrooms, microalgae or seaweeds and for pressing extraction of fruits juices (apples, grapes…) or oils (olive, rapeseed) (79, 96, 109, 111–114). The quality of products (purity, color, texture, flavor, and nutrient) extracted from solid foods (sugar beets, apples, grapes…) and quality of proteins and polysaccharides extracted are less degraded by PEF than by conventional mechanical or chemical pretreatment (108, 110). More recently, several studies pointed to the potentialities of PEF processing for valorization of waste and by-products from agricultural and food processing and for development of sustainable biorefineries (17, 115). Particularly, PEF pretreatment was shown to be more efficient than conventional techniques to extract high-value compounds (phenolics compounds, anthocyanins, carotenoids, pectins, essential oils…) from by-products of artichoke, blueberry, grape, flaxseed hulls, citrus, Norway spruce, alfalfa, rapeseed stem (52, 113, 115–117)…. In many cases, PEF treatment even in combination of mechanical or solvent processing, represents a “greener” extraction processing, compared to conventional techniques, due to higher extraction yield, lower energy consumption and reduced utilization of toxic solvents.

From an energetic point of view, the energy consumption for production of damaged plant tissues varied from 2 to 4 kJ/kg for red beetroot or sugar beet, 6 kJ/kg for sugar beet or around 16 kJ/kg for potato (52), that is significantly lower than conventional methods, such as mechanical processing (20–40 kJ/kg), enzymes (60–100 kJ/kg) or heat treatment (>100 kJ/kg) (15). However, for hard or resistant materials (lignocellulosic biomass) higher electric fields (up to 20 kV/cm) and energies (up to 800 kJ/kg) are required as compared to soft tissues. For juice and oil extraction, in addition to lower energy requirements for tissue disintegration, PEF application provides a possibility to reduce energy required for fruit juice pressing (15). The economic cost for PEF treatment necessary to recover valuable compounds from different matrices (0.1–0.5 euros/ton for chicory, grape skin, fennel, red beetroot, soybean or sugar beet) was estimated to be significantly lower as compared to enzymatic one (7.5 euros/ton) (116). In the same way, Toepl and Heinz (2011) estimated that the total cost (considering the electricity cost, equipment investment, maintenance cost, labor rent, water…) for apple pulp PEF processing was around 2.69 euros/ton as compared to 8.50 euros/ton for enzymatic maceration (118). This way, PEF represents an economically profitable and sustainable alternative and allow the production of juices and extracts presenting higher quality attributes.

From an engineering point of view, large scale biomass PEF-processing devices (up to 50 t/h) have been develop for industrial applications. For example, an industrial system to enhance yield of cloudy apple juice is operated in a German fruit juice company at a 10 t/h scale. PEF pretreatment of potatoes to improve cutting is currently used in 40 French fries companies (115). Currently, the main suppliers of PEF installations for pasteurization or extraction applications are PurePulse (Netherlands) Pulsemaster (Germany), DTI/Elea (Germany), Scandinova (Sweden), Steribeam (Wek-Tec, Germany).

### Membrane Processes

Strictly speaking, single membrane operation cannot be considered as an extraction process. However, combinations of different membrane processes or integration of a membrane operation in an existing industrial process helps to achieve the recovery of valuable compounds from food waste or raw materials. The main successful applications of membrane processes which allow for an increase in the sustainability and the profitability by a more rational utilization of the raw material are undoubtedly in the dairy industry for the production of partially demineralized whey on the one hand, and the production of lactose on the other hand.

Whey is actually the main by-product of dairy industries; it can be obtained during the traditional cheese making processes or as a result of the implementation of ultrafiltration for the treatment of milk to produce cheese or to perform milk standardization (UF whey permeate) as well as for its use to prepare whey protein concentrates (WPC) and whey protein isolates (WPI) (119). Whey obtained during cheese making processes are generally used to produce whey powder. According to cheese making processes, some wheys can contain high amounts of salt which can alter nutritional and food functional characteristics of the powder. Consequently, the value of whey powders increases when salt concentration is reduced and especially when monovalent cations are removed before drying. In contrast to chromatography generally used for demineralization, NF appears to be a more suitable process to achieve this objective since it ensures a simultaneous pre-concentration and partial demineralization of the product. Indeed, NF membranes are able to retain the valuable compounds of whey, such as protein and lactose as well as multivalent ions (i.e., calcium) while monovalent ions are removed in the permeate. Román et al. reported that the efficiency of the process increases when NF is operated in diafiltration mode (120). Depending on their protein contents UF whey permeates can be uses to prepare WPC and WPI. These processes involve at least one or more UF steps which produce permeate with high lactose content (121). The recovery of this lactose is possible thanks to a NF step (122). According to da Siva et al., who studied different combinations of integrated production of whey protein concentrate and lactose derivatives the recovery of lactose increases considerably the economical attractiveness of plants producing WPCs (123).

Although the labeled products derived from food waste are still rather limited and concern mainly dairy by-products (i.e., WPI, lactose), numerous studies have reported the potentialities of membrane processes for the recovery and reuse of food by-products. The combination of UF and NF steps appears to be an efficient purification and concentration process for recovering bioactive peptides from fish protein hydrolysates (124, 125). The separation efficiency of the isolation of charged compounds from hydrolysates containing other neutral solute of similar size can even be further improved by coupling NF and electrodialysis with ultrafiltration membrane (EDUF) (electrically-driven process) in a same process line thanks to the synergy of both driving force (pressure and electrical potential) (126). More recently, there is a growing interest for the production of purified extracts of phenolic compounds involving membrane technologies and in particular NF (127–129). Basically, after the extraction step a two-step membrane separation process has been used, starting with ultrafiltration to remove larger molecules (proteins, polysaccharides and other impurities) followed by nanofiltration for further purification (elimination of salts) and concentration. Sometimes, a microfiltration step is carried out prior to UF step and the NF step is replaced or completed by a reverse osmosis step. However, despite the proven qualities of extracts obtained, investigations are needed in order to guarantee the success of these processes at the industrial level, particularly regarding their performances limited by solute-membrane interactions (130). Further research should focus on the development of membrane materials as well as on process design to obtain high transfer rates and higher selectivity.

## Water Recovery And Food Waste Management

Beside improvement of processing efficiency by significant decreases of energy consumption, process intensification also aims to reduce waste generation and promote a more rational use of natural resources, in particularly water. Indeed, many countries (i.e., China, India, Middle East countries, etc.) have to face to water scarcity which renders the re-use or recycling of water essential for economic and environmental reasons (131). Food industry is one of the most water-consuming industries; it requires large amount of high-quality water for food manufacturing but also water for cleaning purposes, transportation or heat-exchanges. In addition, food processing generates high volume of pollutant wastewaters. These wastewaters are generally non-toxic but their characteristics (chemical oxygen demand COD, minerals, suspended solids, etc.) vary with their origin (type of food industry) and their usage. Even if the re-use of water at the food processing stage is difficult to envisage for sanitary reasons, Beneduce et al. demonstrated that it could be used for irrigation of agricultural crops without significant increase of potential health risk related to microbial quality (132). Moreover, recycling and/or reconditioning water offer the possibility to decrease of environment and water footprints of food processing industry by saving up to 60% of the total water usage (131).

The use of non-thermal technologies discussed above, and in particular membrane separation processes, can help to achieve this goal since they permit the concentration of waste organic matter. Introduced 30 years ago in water treatment processes, membranes separation processes are currently widely implemented as secondary treatment (namely membrane biological reactor (MBRs) which combines biological reactors with membrane filtration units) and tertiary advanced treatments (mainly NF or RO operations) for urban wastewater (133), as well as for decontamination of agro-food industries wastewaters (134, 135). More recently, the co-management of domestic wastewater and food waste in an anaerobic membrane bioreactor (AnBMR) appears to be a potentially attractive strategy since it permits high COD removal and a net positive energy balance of the process due to a simultaneous methane production (136, 137).

In addition, pressure driven membrane processes including MF, UF and NF were successfully used to recover the functional characteristics of soda cleaning in place (CIP) solutions which can thus be re-used (138, 139). Salehi et al. reported that NF permits the reconditioning of colored brines used for ion-exchange regeneration in the sugar industry (140).

The major drawback of membranes processes, irrespective to the technology considered, is membrane fouling which reduces the permeation flux and thus increases the operational cost. This problem has been widely investigated for years, and among the studied strategies, PEF has also been tested for control of biofouling, disinfection of domestic or waste waters (141, 142), or for the pre-treatment of sludges produced during wastewater treatment (15, 143–145). In this last application, it has been shown that PEF facilitates the disintegration of microorganisms and consequently improves the bio-availability of organic carbon, biogas production, solids removal, and sludge quality after anaerobic digestion. For example, a sludge reduction of 27–45% was achieved after implementing electrical treatment to the return activated sludge at 1,650 kJ/kg TSS (146). However, since PEF treatment in wastewater treatment has only been applied at lab or pilot-scale, the energy efficiency of this non-thermal technique can only be estimated, and should be optimized technologically and economically for further industrial implementation.

## Conclusion

High hydrostatic pressure (HHP), dynamic high pressure (high pressure of homogenization, HPH, and ultra-high pressure of homogenization, UHPH), carbon dioxide treatment, PEF, and membrane processes are non-thermal technologies. For decades, they have been investigated for food applications for the purpose of food product stabilization and extraction, as alternative processes to conventional application of thermal treatment, and/or the use of non-eco-friendly solvents. All of them allow for minimal-processed food with improved quality attributes compared to their thermal counterparts. In the same way, membranes and PEF already have a long research story for the treatment of industrial wastewaters, allowing for significant recoveries and thus wastage reduction. Therefore, these technologies are considered promising for the present and future development of sustainable food applications. However, the progress made on the research and the results obtained for their industrial implementation differ from one technology to another, making a direct comparison of these processes difficult. Most of these non-thermal processes are currently applied at a lab or pilot scale, thus inducing higher production cost than large scale industrial thermal devices. Furthermore, recent studies have pointed out the need to consider the overall environmental, economic and social impacts (i.e., energy balances, LCA, waste production/reduction, cost of production, quality of improving standards of living…) of the implementation of these “novel” technologies throughout the food chain. Thus, further cross-sectional investigations should be done to really determine the potentialities and limits of non-thermal technologies to improve the sustainability of food processing operations, and to identify for each “novel” technology, the steps to be optimized to make them more sustainable.

## Author Contributions

All authors listed have made a substantial, direct and intellectual contribution to the work, and approved it for publication. The main editorial contributions of each author are LP-P: high hydrostatic pressure and pulsed electric fields; DC-L: High pressure homogenisation; CC and SM: Carbon dioxide processes; M-PB: Membrane processes.

### Conflict of Interest Statement

The authors declare that the research was conducted in the absence of any commercial or financial relationships that could be construed as a potential conflict of interest.
